# Transplantation of autologous mesenchymal stromal cells in complete cervical spinal cord injury: a pilot study

**DOI:** 10.3389/fmed.2024.1451297

**Published:** 2024-09-12

**Authors:** Carolina Thé Macêdo, Bruno Solano de Freitas Souza, Cristiane Flora Villarreal, Daniela Nascimento Silva, Kátia Nunes da Silva, Clarissa Lima e Moura de Souza, Daniela da Silva Paixão, Milena da Rocha Bezerra, Antônio Olímpio da Silva Moura Costa, Eduardo Santos Brazão, Josildo Pereira Marins Filho, André Costa Matos, Ricardo Ribeiro dos Santos, Milena Botelho Pereira Soares

**Affiliations:** ^1^Gonçalo Moniz Institute, Oswaldo Cruz Foundation (FIOCRUZ), Salvador, Bahia, Brazil; ^2^Hospital São Rafael, Salvador, Bahia, Brazil; ^3^SENAI Institute for Innovation in Advanced Health Systems, SENAI CIMATEC, Salvador, Bahia, Brazil; ^4^Center for Biotechnology and Cell Therapy, Hospital São Rafael, Salvador, Bahia, Brazil; ^5^Faculty of Pharmacy, Federal University of Bahia, Salvador, Bahia, Brazil; ^6^National Institute of Science and Technology for Regenerative Medicine, Rio de Janeiro, Rio de Janeiro, Brazil

**Keywords:** stem cell, spinal cord injury, cellular therapy, mesenchymal stromal cells (MSC), tetraplegia

## Abstract

**Objective:**

Spinal cord injury (SCI) is a serious condition that can lead to partial or complete paraplegia or tetraplegia. Currently, there are few therapeutic options for these conditions, which are mainly directed toward the acute phase, such as surgical intervention and high-dose steroid administration. Mesenchymal stromal cells (MSC) have been shown to improve neurological function following spinal cord injury. The aim of the study was to evaluate the safety, feasibility, and potential efficacy of MSC transplantation in patients with cervical traumatic SCI.

**Methods:**

We included seven subjects with chronic traumatic SCI (> 1 year) at the cervical level, classified as American Spinal Cord Injury Association impairment scale (AIS) grade A. Subjects received two doses of autologous bone marrow derived MSC, the first by direct injection into the lesion site after hemilaminectomy and the second three months later by intrathecal injection. Neurologic evaluation, spinal magnetic resonance imaging (MRI), urodynamics, and life quality questionnaires were assessed before and after treatment.

**Results:**

Cell transplantation was safe without severe or moderate adverse effects, and the procedures were well tolerated. Neurological evaluation revealed discrete improvements in sensitivity below the lesion level, following treatment. Five subjects showed some degree of bilateral sensory improvement for both superficial and deep mechanical stimuli compared to the pretreatment profile. No significant alterations in bladder function were observed during this study.

**Conclusion:**

Transplantation of autologous MSC in patients with chronic cervical SCI is a safe and feasible procedure. Further studies are required to confirm the efficacy of this therapeutic approach.

**Clinical trial registration:**

https://clinicaltrials.gov/study/NCT02574572, identifier NCT02574572.

## Introduction

Traumatic spinal cord injury (SCI) is a serious condition that can lead to partial or complete paraplegia or tetraplegia (1). Currently, there are few therapeutic options for these conditions, which are mainly directed toward the acute phase, such as surgical intervention and high-dose steroid administration (2, 3). These conditions cause a high burden for the patients, their families, and society due to the morbidity associated with SCI; thus, it is of great interest to develop new therapies that can induce partial or complete recovery of SCI patients.

Cell therapy has been intensively investigated in recent years as an alternative treatment for SCI (4–7). Mesenchymal stromal cells (MSC) have been extensively tested, as they are an attractive cell type for therapy because of their easy obtention, safety demonstrated by many clinical trials, and the possibility of autologous application. So far, clinical trials performed using MSC have shown the feasibility and safety of MSC administration in SCI patients and have indicated its potential efficacy (8–10).

In previous studies, our group reported safety and neurological improvements associated with autologous bone marrow derived MSC transplanted directly into the lesions of patients with SCI in the thoracolumbar regions (11, 12). We conducted a pilot study including patients with chronic lesions at the cervical level (tetraplegia) to demonstrate the safety and potential efficacy of autologous MSC transplantation in this clinical setting.

## Materials and methods

### Ethics statement

This study was approved by the Ethics Committee of Hospital São Rafael, Salvador, Bahia (CAAE43085715.9.0000.0048), and registered in the Clinical Trials database (NCT02574572). This study was performed with informed consent and followed the ethical guidelines of the Declaration of Helsinki.

### Objectives and outcomes

The main objective of this study was to evaluate the safety and feasibility of autologous bone marrow derived MSC transplantation in patients with chronic traumatic SCI at the cervical level. The safety outcome was measured by screening for deleterious modifications on resonance magnetic imaging, as well as possible side effects and adverse events related to the protocol procedures. The secondary objective was to assess the potential efficacy of the therapy based on neurological improvement in sensory and motor function, pain scores, and urodynamic and evoked potential studies. Functional improvements were measured using the spinal cord independence measure (SCIM) and functional independence measure (FIM). All data were collected personally by the same researchers, with specific forms and validated questionnaires, to enhance the quality of measurements and results.

### Selection of subjects

From the screening of 41 patients, seven volunteers were included in the study, according to the following inclusion criteria: traumatic SCI at the cervical level under C4 for at least, one year; American Spinal Injury Association (ASIA) impairment scale (AIS) grade A; and age ≥ 18 and ≤ 65 years. The exclusion criteria were open SCI, concurrent infectious disease, terminal illness, neurodegenerative disorders, primary hematological disorders, osteopathies, coagulopathies, hepatic dysfunction, other clinical complications that could contraindicate the procedure, use of metallic implants that contraindicate magnetic resonance imaging (MRI), and participation in other clinical trials.

Participants and researchers, those administering the interventions, and those assessing outcomes were not blinded to the study conditions. All data were collected at Hospital São Rafael, Salvador, Bahia, Brazil.

### Isolation of bone marrow cells and MSC culture

Subjects were submitted to Bone marrow aspiration was performed after hematology, blood biochemistry, urine and microbiological evaluations, and screening for infectious diseases (HIV, HTLV, Chagas disease, and hepatitis B and C). Bone marrow aspiration was performed by a hematologist at the outpatient surgery center. The subjects were sedated and monitored by an anesthesiologist. Local anesthesia with 2% lidocaine was performed, and approximately 100 ml of bone marrow was collected from the anterior and posterior iliac crests using a bone marrow aspiration needle with adjustable length (1.0 to 4.8 cm) and 15 gauge (Carefusion, San Diego, CA, USA), and 20 ml syringes containing 1 ml of heparin 5,000 IU (Cristália, Itapira, Brazil).

Syringes containing bone marrow cells were sent to a certified cGMP facility at São Rafael Hospital for processing under standard protocols as previously described (12). The cells from one participant did not meet the established quality criteria; therefore, they were excluded from the study. MSCs were successfully expanded from the remaining six participants for approximately four weeks. Confluent autologous MSCs at passage 3 or 4 were resuspended in 5% dextrose in water solution (278 mmol/L) (B Braun, São Gonçalo, Brazil) at a concentration of 5×10^7^ cells/mL and transferred into 1 mL syringes for administration to subjects. Prior to injection, the cells were characterized by immunophenotyping using flow cytometry analysis, differentiation assays, G-band karyotype analysis, and tested for sterility, mycoplasma, and endotoxin content.

### Quality control

Each batch of cells was subjected to rigorous quality control tests prior to use, including multi-lineage differentiation assays, flow cytometry, and cytogenetic analysis. The criteria for approval of cellular products for transplantation are listed in Supplementary Table 1. Cell viability found in all batches was above 90% in the final product, as evaluated by trypan blue exclusion.

The multipotency of MSCs was confirmed by adipogenic, chondrogenic, and osteogenic differentiation assays using commercially available kits, following the manufacturer’s recommendations (StemPro adipogenesis, chondrogenic, and Osteogenesis Differentiation Kits, GIBCO). Histochemical staining was used to evaluate cell morphology in differentiated cultures, and oil red was used to visualize lipid inclusions (adipocytes), alcian blue for glycosaminoglycans (chondrocytes), and alizarin red for visualization of mineralized matrix (osteoblasts). Images were captured using an AX70 optical microscope (Olympus).

For immunophenotyping, MSCs were dissociated using 0.25% trypsin solution (ThermoFisher Scientific), washed with PBS, and incubated for 30 min at 4°C with the following antibodies: FITC anti-CD11b, PerCP anti-CD45 (Beckman Coulter, Brea, CA, USA), PerCP anti-CD73, PE-Cy5 anti-CD117, APC anti-CD90, APC anti-CD44 (BD Pharmingen, San Diego, CA, USA), and FITC anti-CD105 (R&D Systems, Minneapolis, MN, USA). Acquisition and analysis were performed using an LSR Fortessa cytometer with FACSDiva software (Becton Dickinson, San Jose, CA, USA). A minimum of 10,000 events were recorded.

Cytogenetic evaluation was performed using G-band karyotyping prior to transplantation to detect possible structural and numerical alterations in chromosomes induced by *in vitro* expansion. MSCs were treated with 16 μg/ml colchicine (Cultilab, Campinas, Brazil) for a period of six hours. The cells were trypsinized, resuspended, centrifuged, exposed to hypotonic solution of 0.075 M KCl, placed in a water bath at 37°C for 30 min, and fixed with Carnoy’s solution 3:1 (acetic acid / methanol). GTG banding was performed by incubating slides at 60°C overnight, followed by treatment with 0.1% trypsin and Giemsa staining. Analyses were performed in 20 cells for each passage by observation under a BX61 microscope (Olympus) coupled to a digital imaging system (Applied Spectral Imaging, Carlsbad, CA). Analyses were performed in accordance with the international classification ISCN (An International System for Human Cytogenetic Nomenclature).

All samples were tested for sterility using the BactABACT/ALERT^®^ system (Biomerieux), for endotoxin content using the Endosafe^®^ system with 0.05–5.0 endotoxin units/mL (EU/mL) sensitivity cartridges provided by Charles River, and for Mycoplasma sp. contamination using the Mycoalert^®^ kit (Lonza).

### Cell transplantation and follow up

The treatment consisted of two administrations of MSCs. First, participants underwent a hemilaminectomy for direct injection of the cells into the lesion area, delivering 5×10^7^ cells resuspended in 1 mL saline solution. Subjects received a second injection of MSC (5×10^7^ cells in 1 ml) 3 months later via the intrathecal route, after local anesthesia was done using 2% Lidocaine without vasoconstrictor. The intrathecal administration was performed using a syringe attached to a Pencan PTA pencil 25G / 27G needle, in the lumbar spinal cord, above the S2 level.

Both procedures were performed by a neurosurgeon at the São Rafael Hospital, Salvador, Bahia, Brazil. One patient had delayed cell growth and did not undergo cell transplantation.

Clinical and neurological assessments were performed for one year and, at each follow-up, complete clinical, neurological, and AIS scale assessments were conducted. Urodynamic studies were performed before and 3rd, 6th and 12th months after the first cell administration. MRI was performed before transplantation and at the end of the follow-up period.

### Clinical pain measures

All pain measurements were performed as previously described (11) in a quiet room with the temperature maintained between 21 and 23°C. At the time of testing, the subjects rated their present pain using an unanchored visual analog score (VAS). Data from the VAS scale are presented in millimeters. Next, the subjects were asked to indicate where they were currently experiencing chronic pain by shading in the areas on a drawing of the dorsal and frontal views of the human body. Additionally, subjects were also asked to fill in a standard Brazilian-Portuguese language version of the McGill Pain Questionnaire (13), the results of which were then quantified using the pain rating index (14).

### Sensory assessment

For cutaneous mechanical stimulation, a soft brush (SenseLab Brush 05, Somedic, Sweden) and von Frey monofilament 98 mN (Touch Test Sensory Evaluator, Stoelting, Wood Dale, IL, USA) were used to apply brushing and touch stimuli, respectively, as previously described (11). The test sites were identified based on anatomical landmarks to ensure that the same site could be accurately located in subsequent sessions. For all participants, the starting stimulation site was the C2 dermatome (the key point for sensory testing from ASIA), where the sensation was expected to be within normal limits. Participants kept their eyes closed and were instructed to report each mechanical stimulus (brush or touch) they perceived with “yes.” For each trial, a monofilament (98 mN) was applied perpendicular to the skin surface, and once the filament was fully bent, it was held in place for approximately one second before being lifted off the skin. The next area was stimulated, following a positive response. Brush stimuli was applied continually, from the starting site, until the subjects reported a switch from “yes” (could feel the stimulus) to “no” (could not feel the stimulus).

### Urodynamic analysis

A urodynamic study was performed prior to and at 3, 6, and 12 months after MSC transplantation. The following parameters were measured using cystometry: maximum bladder capacity, compliance, bladder sensation, presence of detrusor overactivity, and presence of urinary incontinence.

Compliance was measured when the bladder showed a filling ability greater than 200 ml in the absence of detrusor overactivity. Bladder sensation was marked as absent, partially preserved, or completely preserved, and non-specific bladder awareness, as previously described (11).

Subjects with micturition were also evaluated using a pressure-flow study. For the urodynamic study, we used the Dynamed Pro-Life Technology, São Paulo, Sp, Brazil dynapack mpx 816 equipment. For cystometry, two plastic urethral probes were inserted into the bladder (6 Fr to measure intravesical pressure and 8 Fr for filling). A 10 Fr rectal probe was inserted to measure intra-abdominal pressure. The filling was performed with distilled water at room temperature at a rate of 40 ml/min. The results are described as medians, minimum, and maximum.

## Results

### Subjects

The six volunteers who received cell transplantation in the study (five males and one female) had a mean age of approximately 40 years. The demographic and neurological characteristics of the participants are presented in Table 1. The participants had chronic traumatic SCI with a mean duration of 128.5 months (range, 24–192 months). All patients were classified as AIS grade A according to the ASIA of Anesthesiologists guidelines and had injuries to the cervical segment of the spinal cord.

**TABLE 1:** Demographic, clinical, and neurological features of the subjects at the time of enrollment.

Subject	Sex	Age (years)	Time after SCI (months)	SCI level	AIS grade
1	Male	34	159	C6-C7	A
2	Male	55	192	C5-C7	A
3	Male	48	84	C6-C7	A
4	Female	37	24	C4-C5	A
5	Male	30	132	C4-C5	A
6	Male	35	180	C5-C6	A

AIS, American Spinal Injury Association impairment scale; SCI, spinal cord injury.

### Safety assessment

Intralesional transplantation of MSCs through hemilaminectomy injection, followed by intrathecal injection three months later, was a feasible and safe therapeutic scheme. The procedures were well tolerated, and no adverse events associated with MSC administration were recorded. None of the participants presented with fever or infection. Nuclear magnetic resonance images obtained before MSC transplantation revealed the presence of spinal cord cavities, myelomalacia, and syringomyelia in most subjects. Cord atrophy areas and gliosis, as well as findings associated with the primary surgery (that is, epidural fibrosis, soft tissue), were found in all subjects. MRI analysis after MSC transplantation revealed no alterations in hyperintense signals, extension of cavities or appearance of new gliosis areas. Moreover, no signs of ectopic tissue formation were observed during the follow-up.

### Clinical assessment

Neurological evaluation revealed discrete improvements in sensitivity below the lesion level following treatment, with no alterations in AIS scores (Table 2). The FIM score showed an increase of three and a decrease in the other three patients, while the SCIM score showed a small increase in five patients at 0 and 12 months after transplantation (Table 2). Clinical measures of pain, obtained using the pain rating index from the McGill Pain Questionnaire and VAS, were performed before and 1, 3, 6, and 12 months after MSC transplantation. Three patients had no neuropathic pain before transplantation and remained pain-free throughout the follow-up period. One participant, without pain at baseline, presented with transient neuropathic pain after transplantation, which had completely disappeared six months post-transplant. One subject with mild neuropathic pain at baseline was pain-free on all post-transplant measures, while one patient who had severe pain at baseline remained unchanged at follow-up.

**TABLE 2:** Summary of outcome measures comparing before treatment (*T* = 0) and 12 months after treatment.

	AIS grade		FIM score		SCIM	
Subject	*T* = 0	12 m	*T* = 0	12 m	*T* = 0	12 m
1	A	A	82	71	26	27
2	A	A	78	115	42	56
3	A	A	50	55	22	23
4	A	A	59	76	24	22
5	A	A	75	61	31	33
6	A	A	76	62	26	34

AIS, ASIA impairment scale; FIM, functional independence measure score; SCIM, spinal cord independence measure; *T*, Time; m, months.

### Sensitivity to mechanical stimulation

Cutaneous mechanical sensitivity was identified for both superficial and deep stimuli by applying a soft brush and a von Frey monofilament (98 mN). Five subjects showed some degree of bilateral sensory improvement for both superficial (Table 3) and deep (Table 4) stimuli compared to the pretreatment profile. Sensitivity gains were maintained or increased 12 months post-treatment compared to the 1-month post-treatment assessments. One subject demonstrated superior sensitivity recovery, exhibiting at the 12-month time point a positive response to mechanical stimuli in up to 11 originally unresponsive dermatomes. However, for one subject, neither deep nor superficial sensitivity improved.

**TABLE 3:** Response to mechanical stimulation–SenseLab Brush 05.

Subject#	Number of dermatomes with positive response to brushing stimulation*		
	Before treatment	After treatment	
		1 month	12 months
1	9/8	10/9	9/9
2	8/8	10/10	10/10
3	8/8	10/9	10/9
4	5/6	10/10	13/17
5	7/5	8/8	10/8
6	8/6	8/7	8/6

*Side of the body (right/left). # means number.

**TABLE 4:** Response to mechanical stimulation—von Frey filaments.

Subject#	Number of dermatomes with positive response to von Frey stimulation*		
	Before treatment	After treatment	
		1 month	12 months
1	5/7	8/8	9/9
2	7/7	10/10	10/10
3	7/7	8/8	8/8
4	5/5	9/10	15/15
5	6/6	8/8	10/10
6	8/6	7/6	7/7

*Side of the body (right/left). # means number.

### Urological evaluation

The maximum cystometric capacity changed from 470 ml (150–740 ml) to 450 (230–500 ml) without statistical significance. Bladder compliance was good preoperatively at 20 ml/cmH20 (15–20) and remained at 20 mL/cmH20 after the procedure (17–20). There were no modifications in bladder sensation, which was classified as absent in all patients pre-and postoperatively. Five subjects presented detrusor overactivity during bladder filling and following MSC transplantation; two of these patients improved, with no OB in the urodynamics 6 and 12 months after the procedure. Throughout the study, all participants had urinary incontinence and required intermittent urinary catheterization.

## Discussion

Therapeutic options available for restoring functional loss caused by complete SCI, such as physical therapy and surgical decompression, result in limited effective and lasting outcomes (3). Several clinical trials are being conducted to determine whether the effects of MSC therapy in animal models can be translated into human SCI (8, 9, 15, 16). Here, we show that MSC administration to patients with complete, chronic cervical SCI is feasible and safe. Since these were the primary outcomes of the study, a population with cervical chronic lesions (2 to 16 years of injury) was selected, similar to previous studies with thoracolumbar chronic lesions (11, 12).

Two different administration routes were used in this study. First, an intralesional injection was performed after a hemilaminectomy procedure (to facilitate the access of transplanted cells by extracting any possible scar tissue at the site), followed by a second injection via the intrathecal route three months later. This two-dose protocol was used based on a previous study by our group, in which most of the beneficial effects were observed within the first three months after MSC transplantation in patients with complete thoracolumbar SCI (12).

The main cell type tested in SCI studies is mesenchymal stem cells (MSC), which can be easily obtained from different cell sources such as bone marrow, adipose tissue, and Wharton’s jelly from umbilical cord (17–20). Many clinical trials have shown the safety of MSC administration in several diseases, including SCI (8, 10, 21, 22). Cell preparation followed the quality control criteria, as shown before (9). Our study reinforces the safety of MSC administration by using a two-dose therapeutic scheme.

In a previous study in a mouse model of SCI, therapeutic effects of MSC administration were seen despite of very few cells alive were found a week after MSC administration into de spinal cord (23). MSCs contribute to tissue repair mainly by the paracrine action of their secretome, which comprises a wide range of immunomodulatory, angiogenic, antiapoptotic, and growth factors, supporting cell survival and tissue regeneration (reviewed in 24). The mechanisms suggested by these studies to promote improvement of spinal cord injuries include: reduction of inflammation and glial scar, remyelination, proliferation of neural precursor cells, and induction of angiogenesis (23, 24). Moreover, in a rat model, the transplantation of MSCs by intrathecal route has been shown to allow the biodistribution to the brain (25). Therefore, the paracrine effect of MSC in the brain inducing neuroplasticity may be explained by a local action of the cells.

In the present study, the participants had SCI lesions in the cervical region. Although this was a pilot study, there was less indication of improvement compared to our previous clinical study, in which participants with SCI in the thoracolumbar region were included (12). In fact, in this previous study, a positive correlation between motor and sensitivity improvements and lower lesion levels was observed, suggesting that MSC treatment of lesions in the cervical region may be less effective. Moreover, the association of cell therapy with a rehabilitation protocol, may contribute to greater gains in motor function, since exercise was shown to induce plasticity and activate pathways involved in motor recovery (26).

Despite the large number of studies conducted in preclinical and clinical settings, there are no established therapies for the treatment of SCI. Although a significant number of SCI patients have already been treated with MSC therapy, the protocols, participants’ profiles, cell therapy products, routes, doses of transplantation, and measured outcomes vary among the studies (9). Therefore, there is a need for large, controlled studies to determine whether this type of therapy will in fact be beneficial for one or more groups of patients with SCI.

## Conclusion

This study suggests that autologous MSC transplantation is a safe procedure for patients with chronic cervical SCI injury. Additional studies are required to determine if this therapy will bring benefits, especially addressing the time of administration post-SCI, therapeutic scheme, and route of administration.

## Data availability statement

The original contributions presented in this study are included in this article/Supplementary material, further inquiries can be directed to the corresponding author.

## Ethics statement

The studies involving humans were approved by the Ethics Committee of Hospital São Rafael, Salvador, Bahia. The studies were conducted in accordance with the local legislation and institutional requirements. The participants provided their written informed consent to participate in this study.

## Author contributions

CM: Conceptualization, Data curation, Formal analysis, Investigation, Methodology, Project administration, Software, Supervision, Validation, Visualization, Writing – original draft, Writing – review & editing. BF: Investigation, Methodology, Supervision, Writing – original draft, Writing – review & editing. CV: Formal analysis, Investigation, Methodology, Supervision, Writing – original draft, Writing – review & editing. DNS: Formal analysis, Investigation, Methodology, Visualization, Writing – original draft, Writing – review & editing. KS: Formal analysis, Investigation, Writing – original draft, Writing – review & editing. CS: Investigation, Methodology, Writing – original draft, Writing – review & editing. DSP: Investigation, Methodology, Project administration, Writing – original draft, Writing – review & editing. MR: Investigation, Methodology, Project administration, Writing – original draft, Writing – review & editing. AS: Investigation, Methodology, Visualization, Writing – original draft, Writing – review & editing. EB: Investigation, Methodology, Writing – original draft, Writing – review & editing. JM: Investigation, Methodology, Writing – original draft, Writing – review & editing. AM: Formal analysis, Investigation, Methodology, Writing – original draft, Writing – review & editing. RS: Conceptualization, Investigation, Methodology, Writing – original draft, Writing – review & editing. MS: Conceptualization, Data curation, Formal analysis, Funding acquisition, Investigation, Methodology, Project administration, Resources, Software, Supervision, Validation, Visualization, Writing – original draft, Writing – review & editing.
