# Supplementary material for: Transplantation of autologous mesenchymal stromal cells in complete cervical spinal cord injury: a pilot study
Source: Front Med (Lausanne). 2024 Sep 12;11:1451297. doi: 10.3389/fmed.2024.1451297 (PMC11424397; doi:10.3389/fmed.2024.1451297)
Supplement: Supplementary file 1 [file Table_1.DOCX]

**Supplementary File**

**Table S1: Quality controls of MSCs preparations.**

| Quality controls | Release criteria |
| --- | --- |
| Sterility testing | Sterile |
| Cell viability | ≥ 80% |
| Flow cytometry analysis | CD90 ≥ 95% CD45 < 2%  CD73 ≥ 95% CD11b < 2%  CD105 ≥ 95% CD34 < 2%  CD44 ≥ 95% CD19 < 2%  CD146 ≥ 95% HLA-DR < 2% |
| Karyotyping | 46, XX or 46, XY |
| Endotoxin testing | < 2 UE/ml |
| Mycoplasma testing | Absent |
| Cell differentiation | Able to differentiate in adipocytes, osteocytes, and chondrocytes |
